# Application of the Champion Health Belief Model to determine beliefs and behaviors of Turkish women academicians regarding breast cancer screening: A cross sectional descriptive study

**DOI:** 10.1186/s12905-019-0828-9

**Published:** 2019-11-06

**Authors:** Nukhet Kirag, Mehtap Kızılkaya

**Affiliations:** 1 Public Health Nursing Department, Aydın Adnan Menderes University Faculty of Nursing, Kepez Mevkii, 09010 Efeler/Aydın, Turkey; 2 Psychiatric Nursing Department, Aydın Adnan Menderes University Faculty of Nursing, Kepez Mevkii, 09010 Efeler/Aydın, Turkey

**Keywords:** Breast cancer, Health belief model, Screening, Barriers, Turkish academicians

## Abstract

**Background:**

Breast cancer is an important cancer type and the most common malignancy among women in both developed and developing countries and the second leading cause of cancer death in women worldwide. This study aimed to examine the projected risk of breast cancer in Turkish women academician, determine the levels of their breast cancer screening behaviors and uncover the relationship between their health beliefs and screening behaviors.

**Methods:**

This cross-sectional descriptive study was conducted from March to July 2018 in the province of Aydın, Turkey with a total of 200 female academicians. The data were collected using questionnaires filled out by the participants and the Turkish version of the Champion Health Belief Model Scale. Data were analyzed using t test, ANOVA, Chi-square and logistic regression performed with Statistical Package for Social Sciences version 20.

**Results:**

The mean age of the female academics was 36.1 ± 0.53 years. The female performing breast self-examination had higher perceived sensitivity (OR = 2.88, 95% Cl 1.32, 2.66) benefits to breast self-examination (OR = 0.90, 95% Cl 0.82, 0.99), self-efficacy (OR = 0.87, 95% Cl 0.81, 0.93) health motivation (OR = 1.74, 95% Cl 0.50, 0.90), benefit to mammography (OR = 0.97, 95% Cl 0.88, 1.08), lower barrier to mammography (OR = 1.05, 95% Cl 1.0, 1.09) than women who did not. Female academics with clinical breast examination had higher self-efficacy (OR = 0.91, 95% Cl 0.86, 0.97) and lower barrier to mammography (OR = 1.06, 95% Cl 1.02, 1.10) than women who did not. The female with take mammography had higher sensitivity (OR = 0.84, 95% Cl 0.72, 0.98), lower barrier to breast self-examination (OR = 1.08, 95% Cl 1.02, 1.15) and lower barrier to mammography (OR = 1.09, 95% Cl 1.04, 1.14) than female who did not.

**Conclusions:**

Female academicians in Turkey exhibit positive attitudes towards breast self-examination, clinical breast examination and mammography as they have higher perceived sensitivity against breast cancer, self-efficacy and fewer barriers. Long-term community-based programs should be extended to different groups of women from a variety of socio-demographic environments.

## Background

Breast cancer (BC) is an important cancer type and the most common malignancy among women in both developed and developing countries [1], and the second leading cause of cancer death in women worldwide [2]. It accounts for 30–40% of all the cancers in women all over the world [3]. Among adolescent and young women, BC ranks as the most frequently diagnosed invasive cancer, and represents approximately 25% of BC cases diagnosed among all women in the United States [4]. In addition, young women diagnosed with BC have a worse clinical course than older women. The incidence of BC in young women also varies by race, with young black women having a much higher incidence compared with white women in the same age group [5]. The incidence rate of BC is also increasing rapidly in Turkey, 45.1 in 100,000 women [6].

Even though the incidence of BC has increased, the death rate has fallen due to early diagnosis and effective treatment [7]. Although the American Cancer Society no longer recommends that all women perform monthly breast self-exams (BSE), all women should become familiar with both the appearance and feel of their breasts and report any changes promptly to their physician [7]. American Cancer Society recommends that women should undergo regular screening mammography starting at age 45 years [7].

Mammography, clinical breast examination (CBE) and BSE are recommended for the early diagnosis of BC [6]. In Turkey, national society-based BC screening is performed by the Family Health Centers (FHC), Cancer Early Diagnosis, Screening and Education Centers (CEDSEC) oversight by the Social Health Centers (SHC). Although the main screening method is mammography, CBE is also performed for every woman who is screened in order to increase the efficiency of mammography. Furthermore, a consultancy service must be offered to every woman over 20 years of age to enable them to perform BSE on their own, to create awareness in the society [6]. According to the national screening standards for breast cancer in Turkey, BSE must be performed once a month over the age of 20; CBE must be performed once in 2 years over the age of 20 and once a year over the age of 40; and mammography must be performed once in 2 years between the ages 40 and 69 [6].

Beliefs have powerful effects on lifestyles. The Champion health belief model is a psychosocial model that is intended to explain health behaviors and to determine the factors that affect women’s BC beliefs and screening behaviors. According to this model, health behavior, which is the integration of individual perceptions and values directing people to certain ends, is directly related to the development of diseases [8]. Education and health beliefs are critical in the early diagnosis of BC in developing countries where the number of female university graduates is lower [8]. Previous studies have shown that most Turkish women do not carry out regular BC screening behaviors in practice [8–10]. New policies regarding BC are constantly being developed in Turkey, which is also considered as a developing country. Thus, one of the goals of the Turkey Cancer Control Plan 2013–2018 is to increase BSE and mammography in asymptomatic women for early diagnosis [11].

The major function of a university is research, education and public service. It is widely accepted that academicians play an effective role in creating health behaviors. Academicians transfer information and interact with a large part of the population [12]. Female academicians are role models for other woman to protect social rights of women, to lead healthier lives and to assume responsibility for their own health. Academicians as are in a position to inform young people about BC risk factors, types of screening practices and thus affect their behaviors in a way that will reduce the risk of BC and mortality rates [12]. However, studies carried out with female academicians regarding this issue are limited [12–14].

The specific aims of this study were to examine the projected risk of BC in female Turkish academicians, determine the levels of their BC screening behaviors and uncover the relationship between their health beliefs and screening behaviors. We also report the findings from backgrounds and educations of these women both in and outside health areas. The results would reveal if the women with education in health would practice, what they preach.

## Methods

### Study design and sampling

This cross-sectional descriptive study was carried out from March to July 2018 with female academicians in Aydın (Aydın Adnan Menderes University), Turkey. The study population was determined to be 156 with the G-power program using an impact size of 0.40, α = 0.05 and power (1-β) = 0.80 at a confidence level of 95%. Their schools were divided into two groups: health care schools and other schools. The number of female academicians from each school was determined using stratified sampling followed by simple random sampling. The following formula was used to determine the sample size.

n: Sample size,

N: Number of units in the population,

Nh: number of units in layer h,

Sh^2^: variance of layer h,D^2^ = (d^2^ / z^2^),

d: The maximum error amount that can be accepted by the investigator or the difference between the sample mean and the population mean,

z: This is the z value in the standard normal distribution table according to the margin of error.

$$ \frac{\mathrm{N}.\sum \left(\mathrm{Nh}.{\mathrm{Sh}}^2\right)\;\mathrm{n}=}{{\mathrm{N}}^2\kern0.5em .{\mathrm{D}}^2+\sum \left(\mathrm{Nh}.{\mathrm{Sh}}^2\right)\;} $$

A total of 200 female academicians were included in the study. Of them, 135 were in the health care field, and 65 were in other fields.

The inclusion criteria were: Women academician, working in Aydın Adnan Menderes University, agreed to participate in the study.

### Data collection and ethics

This study was approved by the Aydın Adnan Menderes University Faculty of Health Sciences Ethics Committee [code number:2018/08]. Permission to carry out the study was obtained from the Rectorate of Adnan Menderes University before the data collection. A validated and reliable self-administered, structured questionnaire was prepared according to the Health Belief Model Scale for BC Screening, developed by Champion 1984 and the validity and reliability of Turkish version as tested by Gozum and Aydin, together with an extensive review of the literature on sociodemographic forms [15, 16]. After obtaining the participants’ written and verbal consent to participate, the study’s purpose and its benefits for women’s health were briefly explained. Academicians included in the study were visited in their schools and all the participants filled out the forms by their own in approximately 15 min.

### Socio-demographic characteristics questionnaire

The questionnaire was developed for this study. And the questionnaire hasn’t been published elsewhere. This questionnaire included 20 questions about the participants’ age, marital status, school field, title, family type, income level, smoking, drinking alcohol, exercise level, chronic disease, mental illness, giving birth, BC screening in the last 6 months, regular BSE, BC history of close relatives, body type, stress control levels, health assessment, eating habits and sleep habits. In addition, the questions “Have you ever done any BSE?” and “Can you perform a regular BSE?” were asked to determine the practice of BSE, with the response options of “yes” or “no”, “Can you mark your sleep habits” was asked to select one of the given expressions to “I would lay out at the same time as the regular time and be careful to sleep at the same time as the previous day”, “Some nights I only sleep for a few hours, except that I regularly sleep”, “My sleep order does not change every day”, “Do you have a chronic disease?”, with the response options of “yes”, “no”.

### The Champion health belief model scale for breast Cancer screening

This scale has been developed by Champion in 1984 and revised in 1993,1997 and lastly in 1999 for the health beliefs concerning BSE and mammography screening of BC, and it was translated into Turkish by a number of researchers and culturally adapted for use with the Turkish population [15, 16]. This study used the Turkish version of CHBMS developed by Gözüm and Aydın (2004). This particular version includes 52 Likert-type items in six subscales: perceived sensitivity, perceived severity, and benefits of BSE, BSE barriers, self-efficacy and health motivation. The participants were asked to rate each item on a five-point scale: 1, I strongly disagree; 2, I disagree; 3, I am undecided; 4, I agree, and 5, I strongly agree. The highest scores on each subscale are: 3–15 for perceived sensitivity, 6–30 for perceived severity, 4–20 for benefits of BSE, 8–40 for BSE barriers, 10–50 for self-efficacy and 5–25 for health motivation. High scores indicate more positive opinions and attitudes towards health for all the subscales except the subscale of BSE barriers, where higher scores indicate more barriers [16]. The Cronbach’s alpha values were: 0.89 for sensitivity, 0.85 for severity, 0.80 for health motivation, 0.86 for BSE benefits, 0.81 for BSE barriers, 0.91 for BSE self-efficacy, 0.73 for mammography benefits and 0.88 for mammography barriers. Permission to use this scale was obtained.

### Data analysis

Data were analyzed using t-test, One-way ANOVA, and Chi-square tests using Statistical Package for Social Sciences (SPSS) version 20.The threshold for statistical significance was *p* < 0.05. This study used percentages, means and standard deviation values as descriptive statistics. In order to determine the preliminary indicators of BSE, CBE and mammography logistic regression was performed with the factors that were found to be statistically significant in bivariate analysis. This analysis used performing and not performing BSE as dependent variables, and age, title, birth, academic field, BSE training, chronic disease and income level as independent variables. Its results determined relative risk (odds ratio, OR) at a 95% confidence interval (CI). The retraction method (Wald) was used as the regression model.

## Results

The response rate was 100% among participants. The mean age of the female academicians was 36.1 ± 0.53 years (minimum:23-maximum:60) and 51.5% were between the ages of 30 and 40. Of the participants, 57% were married, 67.5% were working in health field and 29.5% were assistant professors. Of them, 90% had nuclear families, 54.5% had more income than expenses, and 73% were non-smokers. Among female academicians, 52.5% did not take alcohol, 60% exercised sometimes, 20% had a chronic disease, and 13% had a mental illness. Of the participants, 7% had a family history of BC and 51.5% had given birth. Sixty-seven (33.5%) female academicians reported that they had been screened for BC in the last 6 months. Eighty-three (41.5%) female academicians reported that they performed BSE regularly on a monthly basis. Ninety-seven (48.5%) female academicians said that they have at least one CBE. Sixty seven (33.5%) participants expressed that they have performed at least one mammography. More than 50% of the participants said that they were in good health, 53.5% said they had normal eating habits, and 49.5% said they had regular sleep habits (Table 1).

**Table 1:** Sociodemographic variables and lifestyle behaviors of academicians

Variables	Number	Percent
Age (Mean ± SD)		36.1 ± 0.53
Under 30	45	22.5
30–40	103	51.5
41 and above	52	26
Marital status		
Married	114	57
Single	72	36
Divorced	14	7
School		
Health field	135	67.5
Outside the health area	65	32.5
Title		
Research assistant	73	36.5
Lecturer	43	21.5
Assistant Professor	59	29.5
Associate Professor	18	9
Professor	6	3
Family Type		
Nuclear family	180	90
Extended family	16	8
Single parent family	4	2
Income level		
More than expenses	109	54.5
Equal to expenses	85	42.5
Less than expenses	6	3
Active Smoking		
Yes	54	27
No	146	73
Current Alcohol intake		
Yes	95	47.5
No	105	52.5
Exercise		
Regular	17	8.5
Sometimes	120	60
No	63	31.5
Chronic Disease		
Yes	40	20
No	160	80
Mental ilness		
Yes	26	13
No	174	87
Giving Birth		
Yes	103	51.5
No	97	48.5
BC screening in the last 6 months (BSE/CBE/Mammography)		
Yes	67	33.5
No	133	66.5
Regular performance of BSE		
No	83	41.5
	117	58.5
Have you ever had a CBE?		
Yes	97	48.5
No	103	51.5
Have you ever taken mammography?		
Yes	67	33.5
No	133	66.5
History of BC in first-degree relatives		
Yes	14	7
No	186	93
Body type		
Slim	53	26.5
Normal	124	62
Overweight	23	11.5
Stress control		
I get angry	48	24
I control my stress	128	64
I ignore stress	24	12
Health assessment		
Very good	26	
Good	106	13
Normal	65	53
Poor	3	32.5
Eating habit		1.5
Normal	107	
Junk food	77	53.5
Abnormal	16	38.5
Sleeping habit		8
Regular	99	
Some nights a few hours	64	49.5
Irregular	37	32
		18.5
Total	200	100

^*^BC Breast Cancer, *BSE* Breast Self Examination, *CBE* Clinical Breast Examination

Table 2 shows the participants’ scores on each subscale of the CHBMS: sensitivity, 7.7 ± 2.1; seriousness, 19.3 ± 5.1; benefits of BSE, 15.1 ± 3.3; barriers to BSE, 16.0 ± 5.3; self-efficacy, 22.0 ± 5.5; health motivation, 20.5 ± 2.6; benefits of mammography, 17.0 ± 3.3, and barriers to mammography, 22.6 ± 8.1 (Table 2). Evaluation of the mean scores for different groups and CHBMS subscales shown in Table 2 found that participants between the ages of 30 and 40 had higher scores in the area of perceived BSE barriers, that participants who were under 30 years old had higher scores in the area of perceived mammography barriers and that participants over the age of 41 had higher perceived self-efficacy scores. The analysis found that there was a significant difference within the subscale of sensitivity and school of employment and chronic disease. Table 2 shows significant associations between BSE barriers and income level, sleep habits, regular BSE and at least one BSE. There were some significant differences in income level, sleep habits, BC screening in the last 6 months, BSE training, at least one BSE, regular BSE and at least one CBE. There were relationships between the subscale of self-efficacy and income level, sleep habits, breast cancer screening in the last 6 months, BSE training, at least one BSE, regular BSE and at least one CBE. A relationship existed between the subscale of mammography benefits and income level. The low-income participants’ scores were significantly higher than those of the other income levels (*p* < 0.05). The subscale of mammography barrier scores of those who had not been screened for BC in the last 6 months and those who never had a CBE were significantly higher than those who had. There was a significant association between the subscale of barriers to BSE and barriers to mammography. The women who had mammograms had fewer perceived BSE and mammography barriers than those who did not have.

**Table 2:** Health beliefs scale of breast cancer screening assessment in women academician

Risk factors	Health beliefs scale of BC screening							
Age (years)	Sensitivity	Seriousness	Motivation	BSE (benefits)	BSE (barriers)	Self efficacy	Mammography (benefits)	Mammography (barriers)
Under 30	7.2 ± 2.4	19.4 ± 5.5	19.6 ± 3.2	15.0 ± 3.1	16.6 ± 4.7	21.9 ± 5.2	16.9 ± 2.4	24.0 ± 8.3
30–40	7.8 ± 1.9	19.4 ± 5.0	20.8 ± 2.4	14.9 ± 3.5	16.8 ± 5.6	21.1 ± 5.7	17.0 ± 3.3	23.8 ± 8.2
41 and above	7.8 ± 2.3	19.1 ± 4.8	20.8 ± 2.5	15.6 ± 2.9	13.7 ± 4.8	23.6 ± 4.8	17.3 ± 4.1	18.9 ± 6.5
*P* value	.298	.944	.053	.544	*.002	*.026	.822	*.001
School								
Health field	7.3 ± 2.3	19.0 ± 5.5	21.1 ± 2.2	15.6 ± 3.0	16.0 ± 4.3	23.5 ± 3.9	17.1 ± 3.3	21.6 ± 7.4
Outside the health area	8.0 ± 1.7	18.7 ± 4.2	20.7 ± 2.2	15.3 ± 3.4	16.7 ± 6.2	20.0 ± 5.2	16.5 ± 2.5	24.7 ± 8.3
P value	*.008	.078	.326	.835	.456	*.000	.420	.935
Title								
Lecturer	7.8 ± 2.08	19.6 ± 5.0	20.3 ± 2.8	15.1 ± 3.0	16.9 ± 4.8	21.6 ± 5.4	17.1 ± 2.9	23.9 ± 8.3
Associate	7.4 ± 2.2	18.9 ± 5.2	20.9 ± 2.3	15.0 ± 3.7	14.7 ± 5.8	22.6 ± 5.6	16.9 ± 3.9	20.7 ± 7.5
P value	.068	.584	.066	.613	*.019	.325	.057	.051
Income level								
More than expenses	7.8 ± 2.4	19.6 ± 5.5	20.7 ± 2.4	15.4 ± 2.7	15.7 ± 5.2	23.0 ± 4.4	17.6 ± 2.8	21.8 ± 8.4
Equal to expenses	7.5 ± 1.7	19.2 ± 4.6	20.5 ± 2.5	15.1 ± 3.0	17.1 ± 4.9	21.2 ± 5.2	16.0 ± 3.9	23.7 ± 7.6
Less than expenses	8.5 ± 1.6	16.1 ± 3.6	18.1 ± 7.3	8.6 ± 8.4	8.3 ± 6.5	11.8 ± 13.0	19.0 ± 1.0	28.5 ± 5.3
P value	.577	.260	.080	*.000	*.000	*.000	*.002	*.064
Chronic Disease								
Yes	8.8 ± 1.7	19.6 ± 5.0	20.5 ± 2.7	15.2 ± 2.6	15.5 ± 5.6	22.4 ± 5.2	15.8 ± 3.7	23.9 ± 7.4
No	7.4 ± 2.1	19.3 ± 5.1	20.5 ± 2.6	15.0 ± 3.5	16.1 ± 5.3	21.9 ± 5.5	17.4 ± 3.2	22.2 ± 8.2
P value	*.000	.753	.892	.118	.658	.894	.580	.415
Sleeping habit								
Regular	7.5 ± 1.9	19.1 ± 4.7	20.6 ± 2.5	15.1 ± 3.4	15.1 ± 5.0	20.6 ± 5.9	16.8 ± 3.1	22.9 ± 8.1
Some nights a few hours	8.0 ± 2.1	19.6 ± 5.1	20.5 ± 2.9	15.2 ± 3.2	17.5 ± 5.7	22.6 ± 4.4	17.3 ± 3.1	22.6 ± 8.7
Irregular	7.6 ± 2.5	19.5 ± 5.7	20.1 ± 2.5	14.6 ± 3.4	15.7 ± 5.0	24.0 ± 4.9	16.9 ± 4.1	21.9 ± 7.1
P value	.398	.785	.681	.722	*.016	*.003	.686	.825
BC screening in the last 6 months								
Yes	8.2 ± 2.0	18.3 ± 5.2	20.7 ± 2.4	15.7 ± 2.6	14.6 ± 4.6	23.3 ± 3.9	17.4 ± 3.3	19.5 ± 6.4
No	7.4 ± 2.2	19.9 ± 5.0	20.5 ± 2.8	14.7 ± 3.6	16.7 ± 5.6	21.2 ± 6.0	16.8 ± 3.4	24.1 ± 8.5
P value	.279	.698	.164	*.009	.199	*.021	.453	*.042
BSE training								
Yes	7.7 ± 2.2	18.8 ± 5.1	20.6 ± 2.5	15.6 ± 2.8	15.1 ± 4.8	23.6 ± 3.8	17.5 ± 2.9	20.7 ± 7.2
No	7.5 ± 2.1	20.3 ± 4.8	20.3 ± 2.9	14.1 ± 3.9	17.6 ± 5.9	18.7 ± 6.7	16.2 ± 4.0	26.2 ± 8.6
P value	.459	.283	.688	*.035	.724	*.000	.151	.523
Have you ever done BSE?								
Yes	7.5 ± 2.1	19.0 ± 5.0	20.7 ± 2.7	15.3 ± 3.5	15.1 ± 5.1	22.8 ± 5.1	17.1 ± 3.6	21.9 ± 8.2
No	8.2 ± 2.0	20.4 ± 5.4	20.0 ± 2.3	14.1 ± 2.4	19.3 ± 4.9	18.9 ± 5.7	16.7 ± 2.0	25.3 ± 7.0
P value	.351	.882	.273	.467	*.000	*.000	*.019	.227
Does it regularly BSE?								
Yes	8.1 ± 2.0	18.7 ± 4.9	20.8 ± 2.4	15.7 ± 2.8	14.1 ± 5.1	23.4 ± 4.7	16.7 ± 3.9	21.2 ± 7.9
No	7.4 ± 2.1	19.8 ± 5.1	20.3 ± 2.8	14.6 ± 3.5	17.3 ± 5.1	20.9 ± 5.8	17.2 ± 2.9	23.5 ± 8.1
P value	.888	.877	.532	*.036	.515	.100	.138	.601
Were there any CBE?								
Yes	7.8 ± 2.4	19.0 ± 5.2	20.9 ± 2.1	15.5 ± 2.8	15.2 ± 5.1	23.3 ± 4.6	17.0 ± 3.8	20.6 ± 7.5
No	7.6 ± 1.9	19.6 ± 5.0	20.2 ± 3.1	14.7 ± 3.7	16.7 ± 5.5	20.7 ± 5.9	17.0 ± 2.8	24.2 ± 7.9
P value	.463	.376	.152	.051	.605	*.001	.939	*.001
Were there any taken mammography?								
Yes	8.05 ± 2.2	18.8 ± 5.26	20.6 ± 2.5	15.2 ± 2.9	14.5 ± 5.3	23.0 ± 4.6	17.3 ± 3.8	19.3 ± 6.8
No	7.53 ± 2.1	19.6 ± 5.04	20.5 ± 2.7	15.0 ± 3.5	16.7 ± 5.2	21.4 ± 5.8	16.9 ± 3.1	24.3 ± 8.2
P value	.306	.336	.263	.268	**.006**	.050	.072	**.000**

^*^BC Breast Cancer, *BSE* Breast Self Examination, *CBE* Clinical Breast Examination

*p* value is below 0.05

The characteristics of the group that was performing BSE and the group that was not were compared statistically. Table 3 shows significant associations between BSE and age, title, giving birth, BC screening in the last 6 months, BSE training, chronic disease and mental illness (*p* < 0.05). Working area (health or not) and family history of BC were not related with BSE (Table 3).

**Table 3:** Association analysis between variables and performing BSE (*n* = 200)

Characteristics	Performing BSE (*N* = 83)		Not performing BSE (*N* = 117)		Statisitcs
	n	%	n	%	
Age (years)					
Under 30	11	13.3	34	29.1	**X**^**2**^ **= 2.546,** ***P*** **= 0.000**
30–40	37	44.5	66	56.4	
41 and above	35	42.2	17	14.5	
Title					
Lecturer	36	43.4	80	69	**X**^**2**^ **= 13.03,** ***P*** **= 0.000**
Associate	47	56.6	36	31	
Giving Birth					
Yes	53	63.9	50	42.7	**X**^**2**^ **= 8.671,** ***P*** **= 0.003**
No	30	36.1	67	57.3	
BC screening in the last 6 months					
Yes	51	61.4	16	13.9	**X**^**2**^ **= 48.65,** **P = 0.000**
No	32	38.6	99	86.1	
School					
Health field	26	63.4	34	51.5	X^2^ = 1.45, *P* = 0.228
Outside the health area	15	36.6	32	48.5	
BSE training					
Yes	69	83.1	63	53.8	**X**^**2**^ **= 18.55,** **P = 0.000**
No	14	16.9	54	46.2	
Chronic Disease					
Yes	25	30.1	15	12.8	**X**^**2**^ **= 9.62,** ***P*** **= 0.008**
No	58	69.9	102	87.2	
History of BC in first-degree relatives					
Yes	3	23.1	10	76.9	X^2^ = 3.35,
No	80	42.9	105	57.1	*P* = 0.186

*BC Breast Cancer, *BSE* Breast Self Examination, *CBE* Clinical Breast Examination

*p* value is below 0.05

Tables 4 presents adjusted ORs for each subscale performing BSE, CBE, and mammography using logistic regression. Female academicians performing BSE had higher perceived sensitivity (OR = 2.88, 95% Cl 1.32, 2.66), benefits to BSE (OR = 0.90, 95% Cl 0.82, 0.99), self efficacy (OR = 0.87, 95% Cl 0.81, 0.93), health motivation (OR = 1.74, 95% Cl 0.50, 0.90), and benefit to mammography (OR = 0.97, 95% Cl 0.88, 1.08) compared to academicians who did not perform BSE. In addition, participants who perform BSE had lower barrier to mammography (OR = 1.05, 95% Cl 1.0, 1.09) than those who did not. Participants who had CBE reported higher self efficacy (OR = 0.91, 95% Cl 0.86, 0.97) and lower barrier to mammography (OR = 1.06, 95% Cl 1.02, 1.10) than women who did not. Academicians who performed mammography had higher sensitivity (OR = 0.84, 95% Cl 0.72, 0.98), lower barrier to BSE (OR = 1.08, 95% Cl 1.02, 1.15), and lower barrier to mammography (OR = 1.09, 95% Cl 1.04, 1.14) than those who did not (Table 4).’

**Table 4:** Logistic regression analysis of health belief model subscales for performing breast cancer screening

Variable	BSE		CBE		Mammography	
	OR(95%CI)	p	OR(95%CI)	p	OR(95%CI)	p
Sensitivity	2.88 (1.32,2.66)	**.000**	0.90 (0.78,1.04)	.182	0.84 (0.72,0.98) **.030**	
Seriousness	1.05 (0.98, 1.13)	.113	1.01 (0.95,1.08)	.571	1.02 (0.96,1.09)	.445
Barriers to BSE	1.19 (1.10,1.29)	**.000**	1.04 (0.98,1.10)	.140	1.08 (1.02,1.15)	**.008**
Benefits to BSE	0.90 (0.82,0.99)	**.041**	1.00 (0.90,1.11)	.946	1.03 (0.92,1.15)	.557
Self efficiacy	0.87 (0.81,0.93)	**.000**	0.91 (0.86,0.97)	**.008**	0.94 (0.88,1.00)	.074
Health motivation	1.74 (0.50,0.90)	**.000**	0.93 (0.83,1.05)	.305	1.02 (0.91,1.15)	.695
Barriers to mammography	1.05 (1.0,1.09)	**.021**	1.06 (1.02,1.10)	**.001**	1.09 (1.04,1.14)	**.000**
Benefits to mammography	0.97 (0.88,1.08)	.694	1.02 (0.93,1.12)	.558	0.98 (0.90,1.08)	.779

*BC Breast Cancer, *BSE* Breast Self Examination, *CBE* Clinical Breast Examination

*p* value is below 0.05

## Discussion

Our findings show that the practices of BSE, CBE and mammography were 41.5, 48.5 and 33.5%, respectively. This rate has ranged from 27.1 to 42.7% in previous Turkish studies [8–14]. However, these studies have shown that women in Turkey perform BSE at less than the desired level. Iranian women [17], Malaysian women [18], Qatari women [19], Saudi women [20] and Indian women [21] also have similarly low prevalence of screening for the early detection of BC. The results from these countries being close to those of Turkey may be due to similar socio-economic and cultural factors. Cultural factors, modesty and the use of Eastern medicine were shown to be significantly correlated with Korean-American women’s health beliefs and cancer screening behaviors [22].

The percentage of academicians in this study who perform monthly BSE (41.5%) was much higher than the previous Turkish studies conducted with academicians [11–14]. Yılmaz et al. (2011) found that female academicians did the recommended BC screening tests such as BSE, CBE and mammography more than housewives [14]. These results suggested that the educational level has a positive effect on performing BSEs. Ekici and Utkualp (2007) reported that 20.9% academician women performed BSE [23]. Ceber et al. (2009) also found that 27.7% of female academicians performed regular BSE [24]. The most important factor in female academicians’ high frequency of BSE performance may be related to education levels and lower BSE barriers. Previous studies of the factors that affect screening behavior have identified these barriers: lack of information, fear and worries [20], fear of a cancer diagnosis, cost, lack of free time, forgetfulness and embarrassment [19]. Regular BSE ranged for Nigerian women to 54.8% [25] for Indian women to 26% [26] for Iranian women to 14.9% and overall 10.6% of women aged 21–53 years had received mammogram [27], while this percentage was much higher than the mammography practice of 51.5% in our study. Whereas, studies conducted in similar populations, the rate of undergoing mammography ranged from 5.1 to 25% [5, 9, 11–14].

OR (Odds ratio) showed that positive attitude on the sensitivity, self efficacy, health motivation, benefit to BSE scales significantly all increased BSE performance. Sensitivity scale significantly increased mammography practice. Self efficacy scale significantly increased CBE performance. Less barriers lead more likely to the practice of BSE, CBE and mammography. Hence, by using the CHBMS construct, the health care provider can understand the beliefs that may affect the women’s BSE, CBE and mammography practices. Higher scores on all scales except for the barriers indicate positive attitude, as expected screening behavior, while for barriers, a higher score indicates negative attitude. In this study, perceived seriousness was not significantly associated with BSE, CBE and mammography practice. Similarly, in other studies on Turkish women, seriousness has been reported to be a nonsignificant predictor of BSE, CBE and mammography [8, 9, 14].

Our study found correlations between mean CHBMS subscale scores and age, school, income levels, chronic disease, sleep habits, BC screening behaviors, BSE training, performing BSE and CBE behavior. The literature includes few studies with which to compare these findings. Similarly, Demirkan et al. (2011) found that the age and profession affect BSE performance [28]. Fouladi et al. (2013) determined that there is a negative relationship between age and mammography barriers and a direct relationship between age and perceived sensitivity [29]. The current results are similar to those of Fouladi (2013). However, Dündar et al. found that age and educational levels did not affect CHBMS subscale scores [30]. Likewise, Altunkan et al. found that age did not affect women’s CHBMS subscale scores [31].

It is important to demonstrate that the perceived benefits of early diagnosis behavior are greater than the perceived barriers [32]. Low perceived barriers and high perceived benefits are important factors in women’s early diagnosis behavior. The results of logistic regression analysis found four CHBMS variables with significant risk ranges (sensitivity, BSE barriers, self-efficacy, and mammography benefits). This study determined that the women who did BSE had higher perceived sensitivity, fewer perceived barriers and higher self-efficacy than the women who did not. Similarly to this finding, Ceber et al. (2009), Çam and Gümüş (2009) and Yılmaz et al. (2011) also reported that women with high self-efficacy carry out BSE more frequently than women with low self-efficacy [14, 24, 32]. Female academicians’ high self-efficacy and health motivation may be related to their educational and social status in the population.

This study found, like previous studies, that female academicians who do BSE have lower perceived barriers [11–14]. Turkish academicians had less perceived barriers and higher self-efficacy levels [13]. Jordanian women had limited knowledge regarding BC despite the national efforts to promote public awareness about BC and screening methods [33]. This difference may be due to the fact that academicians in Turkish universities are better trained and more informed about BSE practice than Jordanian women or due to cultural differences [33]. In some studies, sensitivity, severity, motivation and benefits were not found to be related to performing BSE [15, 32], but in others, these variables were found to be important preliminary indicators of BSE [13, 32].

It is surprising that there was no difference between the academicians in the field of health and academicians in other fields concerning CHBMS scores and BSE, CBE and mammography. However, individuals who have received health education and specialized in this subject are expected to have higher awareness, motivation, self-efficacy and benefit perceptions about BC screening behaviors and low perceptions about the barriers. It was thought that the similarity of the variables such as education and profession could have an impact on such outcome. In this article, the sociodemographic characteristics of female academicians with high educational level and the relationship between chbm scale and subscales were examined in detail.

The fact that this is a cross-sectional study and not including the longitudinal monitoring of the participants constitute its limitations. The data were collected by self-reporting. Since the frequency of BSE, CBE and mammography are based on subjective memories; the participants may have made mistakes in remembering the past history. The study’s sample consisted of female academicians and thus it cannot be generalized to the wider population of Turkey.

## Conclusion

Female academicians in Turkey exhibit positive attitudes towards BSE, CBE and mammography as they have higher perceived sensitivity against BC, self-efficacy and fewer barriers. But there are still more room for progress. Also, women in health disciplines appear as not practicing what they preach. Further minimizing the barriers towards the screening behaviors can effectively persuade the academician women. Interventions should focus more on the practical implementations. Data on the health beliefs can be used to determine the critical factors that affect BC. Improved health education and implementation of critical strategies should further enhance the performance of BC screening. Long-term community-based programs should be extended to different groups of women from a variety of socio-demographic environments.

## Data Availability

The data sets used and analyzed during the current study are available from the corresponding author on reasonable request.

## Abbreviations

BC: Breast cancer
BSE: Breast self examination
CBE: Clinical breast examination
CHBMS: Champion Health Belief Model Scale

## References

[1] Hajian T, Karimollah SA (2014). Health belief model and practice of breast self-examination and breast cancer screening in Iranian women. Breast Cancer.

[2] Boulos DNK, Ghali RR (2014). Awareness of breast cancer among female students at Ain Shams University, Egypt. Global J Health Sci.

[3] Che CC, Coomarasamy J, Suppayah DB (2014). Perception of breast health among Malaysian female adolescents. APJCP.

[4] Ademuyiwa FO, Gao F, Hao L, Morgensztern D, Aft RL, Ma CX (2015). US breast cancer mortality trends in young women according to race. Cancer.

[5] Reeder-Hayes KE, Meyer AM, Dusetzina S, Liu H, Wheeler SB (2014). Racial disparities in initiation of adjuvant endocrine therapy of early breast cancer. Breast Cancer Res Treat.

[6] Republic of Turkey Ministry of Health, 2015. https://hsgm.saglik.gov.tr/depo/birimler/kanser-db/istatistik/Turkiye_Kanser_Istatistikleri_2015.pdf. Accessed 5 Oct 2017.

[7] Smith RA, Andrews KS, Brooks D, Fedewa SA, Manassaram-Baptiste D, Saslow D (2018). Cancer screening in the United States, 2018: a review of current American Cancer Society guidelines and current issues in cancer screening. CA Cancer J Clin.

[8] Avci IA, Kumcagiz H, Altinel B, Caloglu A (2014). Turkish female academician self-esteem and health beliefs for breast cancer screening. APJCP..

[9] Gençtürk N, Demirezen E, Ay F (2017). Health beliefs of midwifery students at Istanbul University about breast cancer and breast self-examination acknowledgements. J Cancer Educ.

[10] Karadag G, Gungormus Z, Surucu R, Savas E, Bicer F (2014). Awareness and practices regarding breast and cervical cancer among Turkish women in Gaziantep. APJCP.

[11] Açıkgöz A, Çehreli R, Ellidokuz H (2015). Determination of knowledge and behavior of women working at a hospital on breast cancer early detection methods, and investigation of efficiency of planned education. J Breast Health.

[12] Duman NB, Algier L, Pinar G (2013). Health beliefs of the female academicians about breast cancer, screening tests and the affecting factors. Int J Hematol Oncol.

[13] Ceber E, Yücel U, Mermer G, Ozentürk G (2009). Health beliefs and breast self-examination in a sample of Turkish women academicians in a university. APJCP.

[14] Yilmaz M, Guler G, Bekar M, Guler N (2011). Risk of breast cancer, health beliefs and screening behaviour among Turkish academic women and housewives. APJCP.

[15] Champion VL (1984). Instrument development for health belief model constructs. Adv Nurs Sci.

[16] Gözüm S, Aydin I (2004). Validation evidence for Turkish adaptation of Champion's health belief model scales. Cancer Nurs.

[17] Montazeri A, Vahdaninia M, Harirchi I, Harirchi AM, Sajadian A, Khaleghi F (2008). Breast cancer in Iran: need for greater women awareness of warning signs and effective screening methods. Asia Pac Fam Med.

[18] Parsa P, Kandiah M, Zulkefli NM, Rahman HA (2008). Knowledge and behavior regarding breast cancer screening among female teachers in Selangor. Malaysia APJCP.

[19] Bener A, El Ayoubi HR, Moore MA, Basha B, Joseph S, Chouchane L (2009). Do we need to maximise the breast cancer screening awareness? Experience with an endogamous society with high fertility. APJCP.

[20] Amin TT, Al Mulhim AR, Al MA (2009). Breast cancer knowledge, risk factors and screening among adult Saudi women in a primary health care setting. APJCP.

[21] Khokhar A (2012). Breast cancer in India: where do we stand and where do we go?. APJCP.

[22] Eun Y, Lee EE, Kim MJ, Fogg L (2009). Breast cancer screening beliefs among older Korean American women. J Gerontol Nurs.

[23] Ekici E, Utkualp N. Female teaching staff attitudes towards breast cancer. J Breast Hlth. 2007:3136–9.

[24] Ceber E, Turk M, Ciceklioglu M (2010). The effects of an educational program on knowledge of breast cancer, early detection practices and health beliefs of nurses and midwives. J Clin Nurs.

[25] Ogunbode AM, Fatiregun AA, Ogunbode OO (2015). Breast self examination practices in Nigerian women attending a tertiary outpatient clinic. Indian J Cancer.

[26] Kumarasamy H, Veerakumar AM, Subhathra S, Suga Y, Murugaraj R (2017). Determinants of awareness and practice of breast self examination among rural women in Trichy, Tamil Nadu. J Midlife Health.

[27] Shiryazdi SM, Kholasehzadeh G, Neamatzadeh H, Kargar S (2014). Health beliefs and breast cancer screening behaviors among Iranian female health workers. APJCP.

[28] Demirkiran F, Ozgun H, Eskin M, Turk G, Cam R, Ozgun O (2011). Cognition of breast cancer among gestational age Turkish women: a cross-sectional study. APJCP.

[29] Fouladi N, Pourfarzi F, Mazaheri E, Aslı HA, Rezaie M, Amani F (2013). Beliefs and behaviors of breast cancer screening in women referring to health care centers in Northwest Iran according to the champion health belief model scale. APJCP.

[30] Dündar PE, Özmen D, Öztürk B, Haspolat G, Akyıldız F, Çoban S (2006). The knowledge and attitudes of breast self-examination and mammography in a group of women in a rural area in western Turkey. BMC Cancer.

[31] Altunkan H, Akın B, Ege E (2008). Awareness and practice of breast self examination (BSE) among 20-60 years women. J Breast Health.

[32] Çam O, Gümüs AB (2009). Breast cancer screening behavior in Turkish women: relationships with health beliefs and self-esteem, body perception and hopelessness. APJCP.

[33] Othman A, Ahram M, Al-Tarawneh MR, Shahrouri M (2015). Knowledge, attitudes and practices of breast cancer screening among women in Jordan. Health Care Women Int.

